# Assessment of Fiber Bragg Grating Sensors for Monitoring Shaft Vibrations of Hydraulic Turbines

**DOI:** 10.3390/s23156695

**Published:** 2023-07-26

**Authors:** Xavier Sánchez-Botello, Rafel Roig, Oscar de la Torre, Javier Madrigal, Salvador Sales, Xavier Escaler

**Affiliations:** 1 IFLUIDS, Universitat Politècnica de Catalunya, 08028 Barcelona, Spain; xavier.sanchez.botello@upc.edu (X.S.-B.); rafel.roig@upc.edu (R.R.); 2 Floating Power Plant A/S, 4941 Bandholm, Denmark; otr@floatingpowerplant.com; 3 ITEAM, Universitat Politècnica de València, 46022 Valencia, Spain; jamadmad@iteam.upv.es (J.M.); ssales@dcom.upv.es (S.S.)

**Keywords:** hydropower, optical fiber sensors, vibration measurement, fluids, high-spatial-resolution mode shapes, strain measurement, rotating shaft

## Abstract

The structural dynamic response of hydraulic turbines needs to be continuously monitored to predict incipient failures and avoid catastrophic breakdowns. Current methods based on traditional off-board vibration sensors mounted on fixed components do not permit inferring loads induced on rotating parts with enough accuracy. Therefore, the present paper assesses the performance of fiber Bragg grating sensors to measure the vibrations induced on a rotating shaft–disc assembly partially submerged in water resembling a hydraulic turbine rotor. An innovative mounting procedure for installing the sensors is developed and tested, which consists of machining a thin groove along a shaft line to embed a fiber-optic array that can pass through the bearings. At the top of the shaft, a rotary joint is used to extract, in real time, the signals to the interrogator. The shaft strain distribution is measured with high spatial resolution at different rotating speeds in air and water. From this, the natural frequencies, damping ratios, and their associated mode shapes are quantified at different operating conditions. Additionally, the change induced in the modes of vibration by the rotation effects is well captured. All in all, these results validate the suitability of this new fiber-optic technology for such applications and its overall better performance in terms of sensitivity and spatial resolution relative to traditional equipment. The next steps will consist of testing this new sensing technology in actual full-scale hydraulic turbines.

## 1. Introduction

Current EU policy [1] encourages the introduction of intermittent and non-dispatchable energy sources, such as wind and solar power, into the electrical grid. Under this scenario, hydraulic turbines are forced to work for longer periods of time at non-optimal conditions [2]. Several flow instabilities, of both stochastic and periodic nature, such as vaneless space vortex structures or the rotating vortex rope, may occur when operating at these conditions [3]. These deleterious flow phenomena result in several negative effects on hydraulic turbines. For instance, they may decrease the turbine efficiency and its life expectancy [4], as well as result in a resonance if they excite a natural frequency of the shaft train, which, in turn, can accelerate fatigue damage or cause a catastrophic breakdown. For this reason, it is of paramount importance to monitor the dynamic response of hydraulic turbines to ensure safe operation and minimize the risk of unexpected breakdowns. As demonstrated by Karpenko M. et al. [5], an important and useful method for assessing the condition of a turbine is frequency response analysis, in which the recorded vibration data are transformed using fast Fourier transform (FFT) spectrum analysis. By doing so, the dynamic properties of the structure can be investigated and the critical resonance frequencies can be determined.

In order to guarantee that hydraulic turbines work safely during transients and off-design conditions, the EU-funded AFC4Hydro project [6] is developing an active flow control system to mitigate or suppress the deleterious fluid phenomena that can take place under these circumstances, such as the rotating vortex rope. This mitigation is performed by means of two different active subsystems that act depending on the evaluated performance of the turbine assessed by a structural health monitoring (SHM) subsystem. An SHM system needs to measure as much spatially distributed and time-distributed data as possible, i.e., covering different locations within the turbine and monitoring long periods of time. However, the installation of such a complex monitoring system on hydraulic turbines is still a challenge. Hydraulic turbines present both stationary and rotating parts, have submerged subcomponents, and operate in an environment with strong electromagnetic interferences. Under these conditions, classical sensors, such as accelerometers, displacement laser sensors, or strain gauges, present limitations due to their high sensitivity to electromagnetic noise, installation complexity, or poor spatial resolution. On the other hand, state-of-the-art sensors, such as fiber-optic sensors (FOSs), could potentially overtake all these limitations. This technology [7,8], with fiber Bragg gratings (FBGs) as the most used FOS [9] due to their cost-effectiveness [10], is extremely sensitive to temperature and strain variations [11] and may be the ideal candidate for monitoring strains in hydraulic turbines [12,13]. FBGs consist of optical fiber waveguides made of silica and covered by an insulating material that reflects the light of a specific wavelength using an optical filtering device connected to one end. The grating structure permits the passing of all wavelengths of light that are not in resonance with it and reflects wavelengths that satisfy the Bragg condition of core index modulation [14]. The reflected wavelength of the light depends on the spacing of a periodic variation in the refractive index that is present within the fiber core. Hence, distributed measuring points need to be created inside a single FBG array by means of exposing the core of the optical fiber to intense laser light with a periodic pattern to permanently increase the refractive index and measure the strain in those points depending on the reflected wavelength [9]. As stated by de la Torre et al. [15], the advantages of FBG sensors reside in their resistance to humidity and corrosion, compactness, small size, and light weight, immunity to electromagnetic interferences, embedding capacity, and simple experimental setup. Additionally, the presence of several sensors in a single FBG ensures high spatial resolution, which is of paramount interest when monitoring strains along the shafts of hydraulic turbines.

Most applications of FOS technologies are found in civil engineering. For instance, they have been widely used for dam health monitoring through the measurement of the deformation, stress, and leakage of dams [16]. Kronenberg et al. [17] measured the deformation of a dam through a new design of deformation sensors installed inside concrete. Fuhr et al. [18] monitored the health of the Winooski hydroelectric dam in the United States by installing 6.4 km of sensing fibers inside the dam. Henault et al. [19] investigated the stress transfer pattern between the fibers and a dam by comparing the experimental measurements with the results of numerical simulations. Artieres et al. [20] developed a new method for dam crack monitoring by means of installing the sensing fiber into nonwoven geotextiles and locating the damage through the instrumented geotextiles. Despite the extensive literature concerned with FOS applications in civil engineering, their potential in mechanical engineering is still under investigation. De la Torre et al. [15] assessed the performance of FBG sensors submerged in water for long periods of time to validate the potential of this type of technology for hydropower applications. He proposed the use of FBG sensors to determine both natural frequencies and damping ratios of a partially submerged beam using a simplified modal analysis. The results agreed with those determined with more established waterproof electric strain gauges and laser vibrometers. Additionally, the presence of several sensors in a single fiber ensured higher spatial resolution compared to those obtained by classical sensors.

Prior to testing FBG sensing technology in actual hydroelectric turbines, it is still necessary to assess its monitoring capabilities in simple partially submerged rotating structures. In this sense, the current paper aims to validate the use of FBG sensors for potential hydroelectric applications by monitoring the modal response and dynamic response of a shaft–disc assembly when it rotates at several speeds and when the disc is submerged in water under different testing conditions.

## 2. Methodology

In this section, the bespoke test rig of the Barcelona Fluids & Energy Lab (IFLUIDS) built at Universitat Politècnica de Catalunya, BarcelonaTech (UPC), is presented. The experimental setup is detailed, giving special focus to the description of the materials and instruments used for the experimental campaign. Additionally, the methodology to design and perform the experimental campaign together with the analysis tools are described.

### 2.1. Experimental Setup

In order to assess the performance of FBG sensors for monitoring vibration in hydraulic turbines, a more relevant test compared to [15] was necessary. After careful consideration, it was agreed that a potential subject for investigation with FBG in the hydraulic field could be the turbine shaft, as it has a simple geometry and easy access, and it is not directly exposed to fluid phenomena that could potentially damage the installation or the sensors themselves. For this, a turbine shaft–runner structure was simplified to a shaft–disc assembly that was installed in a test rig and connected by a couple of pulleys to an electric motor to rotate it at different speeds. Additionally, a test campaign was designed to measure the shaft dynamic response with several FBG sensors embedded along the shaft–disc structure.

#### 2.1.1. Shaft–Disc assembly

**Figure 1:**
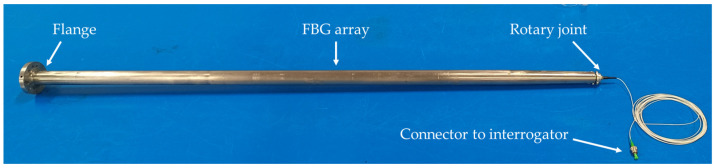
Shaft assembly with the welded flange on the left-hand side and the rotary joint for the FBG sensor on the right.

The construction of the shaft–disc assembly was designed to resemble (yet simplify) a hydraulic turbine rotor. Both the hollow shaft and the disc were manufactured in stainless steel to be able to use them safely both in air and submerged in water. The disc attached to the shaft was 5 mm thick and had a 420 mm diameter with 6 holes (*Φ* = 8 mm) to bolt it to the lower flange of the shaft with 6 M5 bolts. Figure 1 shows a photograph of the instrumented shaft with different elements labeled. The shaft was 1160 mm long and had an external diameter of 30 mm and a wall thickness of 3 mm along the constant cross section. The bottom end had a flange of 10 mm thickness and 70 mm external diameter with 6 M5 holes to attach the disc. The flange was welded to the shaft.

In order to avoid any damage to the FBG array in the radial bearings—shaft contact zone—it was agreed to machine a 2 mm by 2 mm groove along the constant cross section length and install the FBG array inside it.

**Figure 2:**
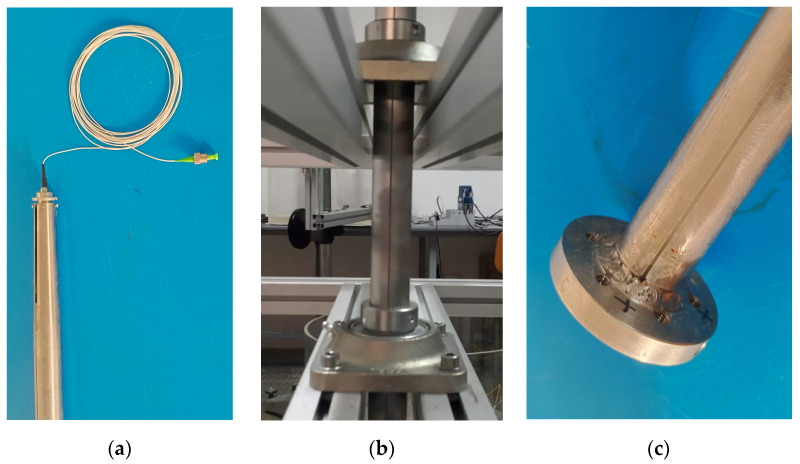
(**a**) Zoomed-in image of the upper part of the shaft where the rotary joint is placed to connect the fiber-optic device with the interrogator; (**b**) groove machined along the shaft to embed the fiber optic and pass it through the bearings; (**c**) zoomed-in image of the bottom end of the shaft where the flange with 5 threaded holes M5 is welded. The 5 mm groove to place the fiber optic can be seen along the shaft in all the pictures.

The upper end of the shaft was carefully machined to progressively reduce the internal diameter along a conic section so as to allow the installation of the fiber-optic rotary joint (FORJ 1.14, from SPINNER), with SPINNER FLEXIFLANGE and 900 µm cable [21]. The joint is formed by two single-mode fiber-optic pigtails with a coating of 900 µm placed inside a housing diameter of 14 mm and classified with IP54 ingress protection. Its compact design is characterized by having a minimum insertion loss value as well as being maintenance-free, which was used to transmit the signal from the on-board rotating system to the off-board stationary interrogator. The model used, presented in Figure 2a, has small dimensions and a weight of 18 g, being able to withstand rotational speeds up to 20,000 rpm. The fiber-optic pigtail connected to this rotary joint is used to transmit the signals from the fiber-optic device to the interrogator. Figure 2b shows, in more detail, the machined groove of the shaft to embed the optical fiber and pass it through the bearings. Finally, Figure 2c shows the bottom end of the shaft, in detail, where the welded flange is located.

#### 2.1.2. FBG Sensor Assembly

**Figure 3:**
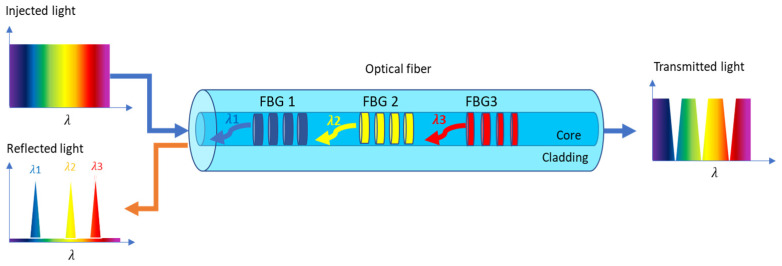
Scheme of three FBG arrays inscribed in standard optical fiber. The reflected wavelengths, λ1, λ2, and λ3, correspond to the Bragg wavelengths of FBG1, FBG2, and FBG3, respectively.

An FBG sensor is a photonic device based on a Bragg reflector, which consists of a periodic perturbation of the refractive index of the fiber-optic core by exposing the fiber to a grating formed by a pattern of laser light fringes. An FBG behaves like a semi-reflective and selective mirror that reflects some wavelengths corresponding to the Bragg wavelength peak and allows the rest to pass through [22]. Bragg wavelength shift tracking can be used to sense temperature and strain variations [23]. Since the Bragg wavelength depends on the grating period, it is possible to multiplex several FBG sensors in the same optical fiber by inscribing gratings with different periods (see Figure 3).

FBG sensors are fabricated by exposing the optical fiber to a pattern of light pulses generated by a UV laser [23]. The ultraviolet light is then diffracted and forced to interfere. In the interference zone, a periodic pattern is created with a period that depends on the angle of interference, photo-inscribing a corresponding grating in the optical fiber. By means of changing the angle of interference, FBGs with different Bragg wavelengths can be created.

To monitor the strain variation along the entire shaft, an array of 19 FBG sensors of 8 mm in length and equispaced 50 mm was fabricated. The fiber optic was then connected directly to one of the four input channels of a Micron Optics sm130 optical sensing interrogator using an FC/APC connector of the fiber-optic pigtail. This interrogator device is equipped with a laser that sweeps from a wavelength of 1510 nm to 1590 nm. Simultaneously, it injects the laser light inside the fiber optic, obtains a spectrum of the reflected light by the FBGs, and computes the peak wavelength of each FBG sensor of the array with a sampling frequency of 1 kHz [24]. Hence, the Bragg wavelengths of the FBGs were designed to be tracked in the interrogator sensing range from 1510 nm to 1590 nm, with a minimum spacing of 1 nm and an accuracy of 5 pm. The Bragg wavelengths of the FBGs were assigned in an increasing and equidistant manner, with 3 nm spacing from 1523 nm (FBG1) to 1577 nm (FBG19).

**Figure 4:**
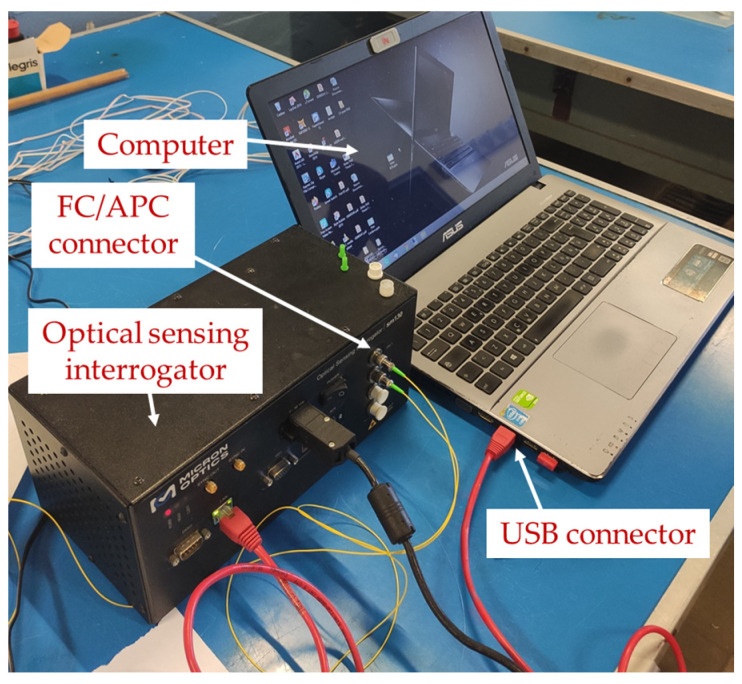
Experimental setup used to acquire the strain measures from the optical fiber using a Micron Optics sm130 optical sensing interrogator and a computer with devoted software.

Figure 4 shows the experimental setup to acquire the strain from the FBG sensors, where the fiber-optic pigtail that comes from the rotary joint of the shaft is connected to a Micron Optics sm130 optical sensing interrogator through an FC/APC connector. Additionally, this interrogator was powered and connected to a computer using a USB connector. Then, devoted software was used to monitor and record the wavelength shift of each FBG sensor.

**Figure 5:**
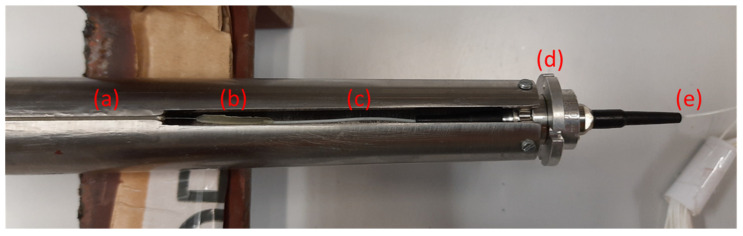
Photograph of the fusion splice between the optical fiber of the rotary joint and the FBG array: (a) beginning of the FBG array, (b) splice made by fusion, (c) optical fiber from the rotary joint spliced to the FBG array, (d) rotary joint, and (e) fiber-optic pigtail of the rotary joint.

In order to install the FBG array inside the groove of the shaft, the FBG array had to be spliced by fusion to one of the optical fibers of the rotary joint as a preliminary step. Figure 5 shows the upper part of the shaft, where the rotary joint was screwed to the tip of the shaft (5d), leaving one fiber-optic pigtail out of the shaft (5e) and another one inside (5c). Then, the FBG array of the shaft (5a) was spliced by fusion to the fiber optic of the rotary joint left inside the shaft using a typical optical fiber splicing machine. Afterward, the spliced part (5b) was coated with a bi-component epoxy resin (ARALDITE^®^ STANDARD ULTRA) to protect it. The wider groove machined next to the joint was used to allow the correct positioning of the rotary joint spliced to the FBG array. The fiber was then placed at the bottom of the groove and a small amount of tension was applied to keep the fiber straight. Once the FBG array was positioned along the shaft, the groove of the entire shaft was also filled with ARALDITE^®^ resin, and it was left to cure for 24 h at room temperature. During the first 40 min of curing, any air bubbles identified in the resin were removed using a needle.

#### 2.1.3. Test Rig

In order to place the shaft in a vertical configuration and rotate it at several speeds, an aluminum Meccano-like reaction frame was built and bolted to a 1500 × 1500 × 20 mm steel plate on the floor to stiffen the assembly. Two ball radial bearings with a diameter of 30 mm were sat bolted on the frame to support the shaft in a vertical position. Additionally, the shaft could rotate in a controlled manner using a DC brushless electric motor with an integrated controller, two steel pulleys attached to the shaft and motor, and a cogged V-belt made of rubber EPDM to transfer the rotation from the pulley of the motor to the shaft. The diameter relation between pulleys guarantees that the shaft rotates at half the angular speed of the motor.

When it was necessary to rotate the shaft–disc assembly in water, a 600 mm tall plexiglass tank with an internal diameter of 492 mm and a wall thickness of 6 mm was used. The tank had a plexiglass lid to avoid water spillage, and the lid had a hole to allow the shaft to pass through it.

#### 2.1.4. Instrumentation

Experimental modal analysis was performed using an impact hammer 086C03 from PCB Piezotronics and by impacting different parts of the shaft while it was still and rotating. The data acquisition of the exerted force by the impact hammer was performed using an IEPE module NI-9231 connected to a cDAQ-9185 chassis from National Instruments.

### 2.2. Test Campaign

The test campaign was designed with a complexity build-up. The campaign aimed to clarify whether the FBG sensors were a good option to study the strain dynamics of the shaft. To do so, the three following objectives were set.

#### 2.2.1. Objective 1: Shaft’s Natural Frequencies and Damping Ratios

For this first objective, the aim was to detect several of the first shaft’s natural frequencies and calculate the associated damping ratios for different shaft configurations. A series of modal tests (for a more detailed explanation of modal tests, see [15]) on the shaft were performed. In particular, the shaft was first tested suspended from a crane using elastic ropes, then it was installed in the test rig in its final position (Position 0) without the disc attached to it and, finally, with the disc. Table 1 summarizes the different conditions studied for this first objective.

These tests should allow us to gain a better understanding of the resolution of the sensors and how the results vary with the location of the sensors on the shaft. This is of special importance in turbine shafts where, very frequently, several sections are not accessible, and one has to install instrumentation in non-optimal locations.

#### 2.2.2. Objective 2: Shaft’s Strain Mode Shapes

**Figure 6:**
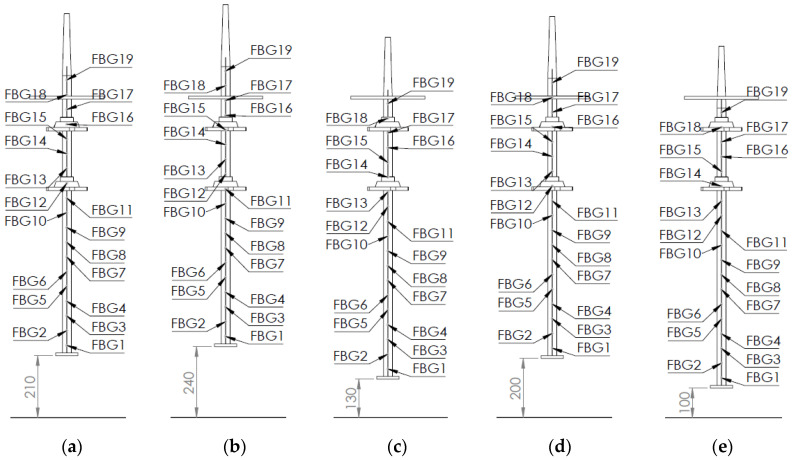
Drawings of the shaft and the position of the different FBG sensors placed along the shaft line. (**a**) Position 0, (**b**) Position 1, (**c**) Position 2, (**d**) Position 3 and (**e**) Position 4. Dimensions in mm.

For the second objective, the aim is to use the high spatial resolution given by the 19 sensors installed over the 90 cm length of the shaft to capture some of the shaft’s bending strain mode shapes. A mode shape is basically the shape a body takes while vibrating at a given natural frequency. Therefore, it graphically represents the motion of the particles of the body. A strain mode shape graphically represents the strain levels of the particles instead. Two changes in the boundary conditions of the shaft were performed for these tests. These changes were more relevant from a hydropower perspective than those in Objective 1, and this is why they were studied. The first one was to study the influence of the relative position of the bearings with respect to the shaft. To do so, upper and lower shaft positions were defined. The upper position (Position 1) was with the shaft–disc assembly installed in the test rig with the lower disc surface placed at approximately 240 mm from the floor. The lower position (Position 2) was similar to Position 1, but the shaft was pushed down through the bearings (and the pulley), leaving the upper disc surface at approximately 130 mm from the floor. It is important to mention that the bearing-to-bearing distance was left unchanged. Figure 6 shows the drawings of these two positions with the different FBG sensors placed on the array.

The second change in boundary conditions to be studied was the influence of water, so the plexiglass tank of water was filled with a fixed volume of water to completely submerge the disc (for both positions) and partially submerge the shaft. Under these conditions, a set of modal tests were performed to obtain the dynamic behavior of the shaft.

See Table 2 for a summary of the test conditions to study Objective 2.

These tests should allow us to show how the shaft strains at each natural frequency and how the boundary conditions affect the strain mode shapes. Knowing the modes of vibration of a turbine shaft during operation is extremely important to understand the orbits of these sections and reduce the risk of localized fatigue problems.

#### 2.2.3. Objective 3: On-Board Monitoring in Rotation

For the third objective, the aim was to take advantage of having an on-board instrument to detect the features of shaft dynamics during rotation. The dynamics of a rotating shaft have some well-known singularities; in particular, there is an apparent change in the shaft’s natural frequency [25] that the authors were very interested to see if it was captured by the FBG array. The change in frequency associated with rotating bodies can be calculated with the following equation:

(1) $$f_{i}\pm \Omega_{s},$$

where $f_{i}$ is the *i*-th shaft’s natural frequency without rotation and $\Omega_{s}$ is the rotational speed. Elnady et al. [26] already showed the split natural frequency of a rotating shaft measured with an on-board accelerometer. Therefore, the shaft’s natural frequency detected with an on-board FBG should also appear as two new frequency peaks from the on-board reference: one that increases with the rotational speed and one that decreases.

**Figure 7:**
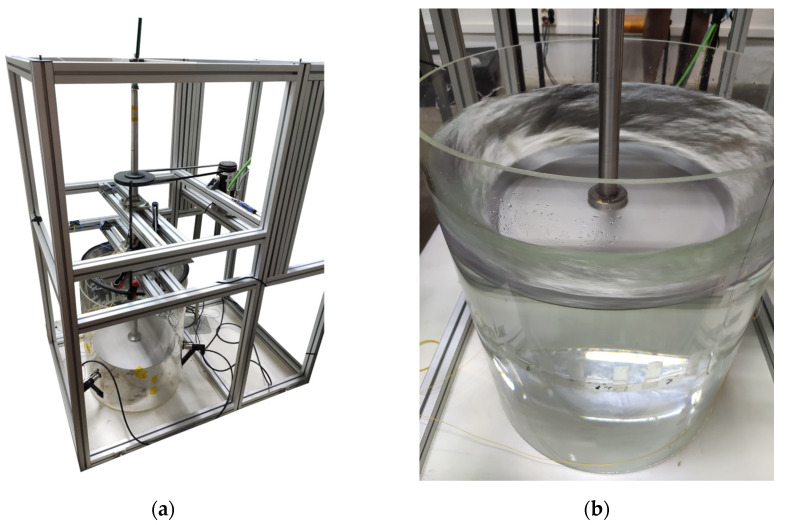
Pictures of the shaft installed in the test rig with the disc attached to the bottom-end flange: (**a**) air, without rotation; (**b**) water, with rotation.

To test this behavior, the shaft was first rotated at different fixed velocities in air and partially submerged in water. For each velocity, a set of modal tests was performed to see if the FBG sensors were able to capture differences in the strain field. The shaft was also monitored during transient operations by increasing or decreasing the rotating speed at a constant rate. Figure 7 shows the shaft installed in the test rig with the disc attached to it both in air and while rotating in water.

For the third objective, a summary of the test conditions is presented in Table 3.

From a hydropower perspective, this effect is also very important to consider when in operation because this is the cause of multiple resonance phenomena. Resonance typically occurs when a given shaft’s natural frequency meets the rotating speed (or a harmonic), producing a significant increase in vibration levels. Therefore, being able to monitor natural frequencies or detect these resonances early enough could reduce failures and costs.

### 2.3. Data Analysis

There is a large variety of identification techniques to analyze modal tests and obtain modal parameters such as natural frequencies and damping ratios [27]. From more simple visual ones to complex methods where parametric models are built and fitted to test data. There are methods that work in the time domain and methods that work in the frequency domain. Among the many options, the Eigensystem realization algorithm (ERA) was selected. The method was first developed by NASA for modal identification and model reduction of dynamic systems from test data [28]. Since then, the technique has been successfully used in many types of dynamic tests, and it can also be modified to work in output-only tests, such as those using ambient vibration [29]. ERA is a time-domain technique that relies on the singular value decomposition (SVD) to formulate the model.

If we assume that the dynamic system can be represented by a state space system:

(2) $$\left\{\begin{array}{c} x_{i+1}=Ax_{i}+Bu_{i}\\ y_{i}={Cx}_{i}+{Du}_{i} \end{array}\right.,$$

with 0 initial conditions and an impact (impulse force) at t = 0, Equation (2) can be simplified to:

(3) $$\left\{\begin{array}{c} \begin{array}{c} x_{i+1}=Ax_{i}+Bu_{i}\\ y_{i}={Cx}_{i} \end{array}\\ u_{0}=1\\ x_{0}=0\; \end{array}\right.,$$

where x is the vector of states; u is the system inputs and y the outputs; and A, B, and C are the Markov parameters, which are system-dependent and from which the modal parameters are extracted. The ERA algorithm starts by building a Hankel matrix from the different sensors’ outputs, which has the following form:

(4) $$H\left(i\right)=\left[\begin{array}{ccc} \begin{array}{cc} y\left(i+1\right) & y\left(i+2\right)\\ y\left(i+2\right) & y\left(i+3\right) \end{array} & \cdots & \begin{array}{c} y\left(i+k\right)\\ y\left(i+k+1\right) \end{array}\\ \vdots & \ddots & \vdots \\ \begin{array}{cc} y\left(i+n\right) & y\left(i+n+1\right) \end{array} & \cdots & y\left(i+k+n\right) \end{array}\right]$$

In each time step, all sensors’ outputs are collected in vectors forming the Hankel matrix. The SVD of the Hankel matrix, H(0), results in two new matrices that, together with the shifted Hankel matrix, H(1), let us estimate the modal parameters [28]. Additional details of the ERA algorithm to extract the modal parameters can be found in [30].

#### Postprocessing Workflow

**Figure 8:**
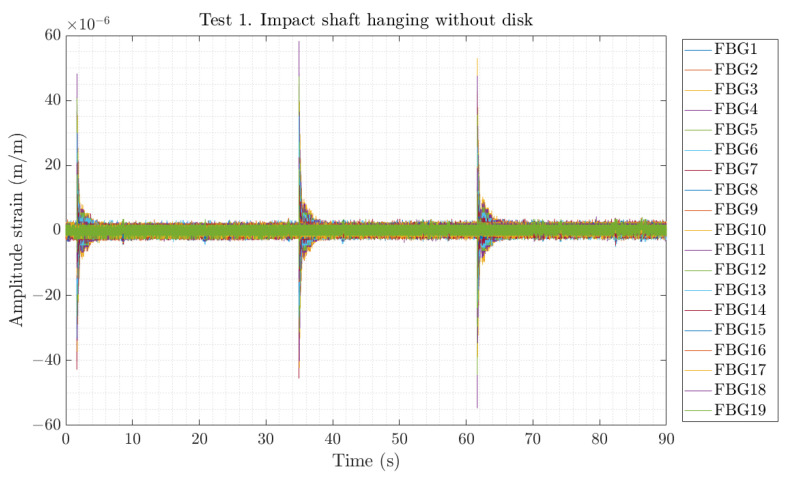
Example of strain time series of three impacts during Test 1.

**Figure 9:**
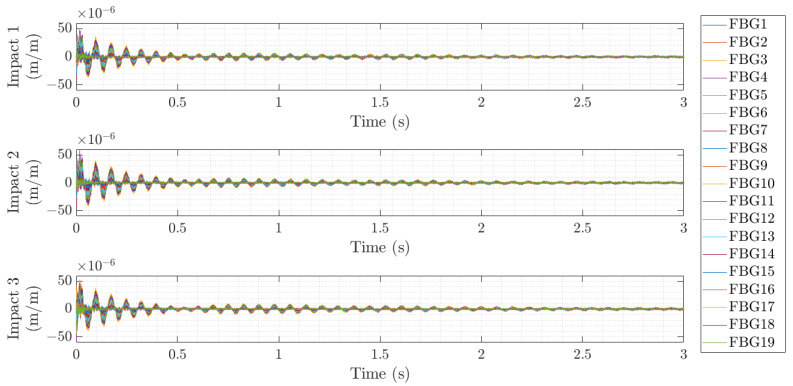
Example of the three extracted impact time series with 3 s free decay response.

The modal tests were performed by hitting with an instrumented hammer in a fixed location of the shaft or shaft–disc assembly. In order to ensure good repeatability of the results, each point was impacted 3 to 5 times, and the structural response of the 19 FBG sensors placed along the shaft was acquired with a sampling rate of 1000 Hz. Figure 8 shows an example of the temporal response obtained by all the FBG sensors when the shaft was impacted in the first test (hanging without the disc). Then, postprocessing of the data was carried out to identify each impact point and cut the measured structural response with a fixed free decay response of 3 s, as shown in Figure 9.

**Figure 10:**
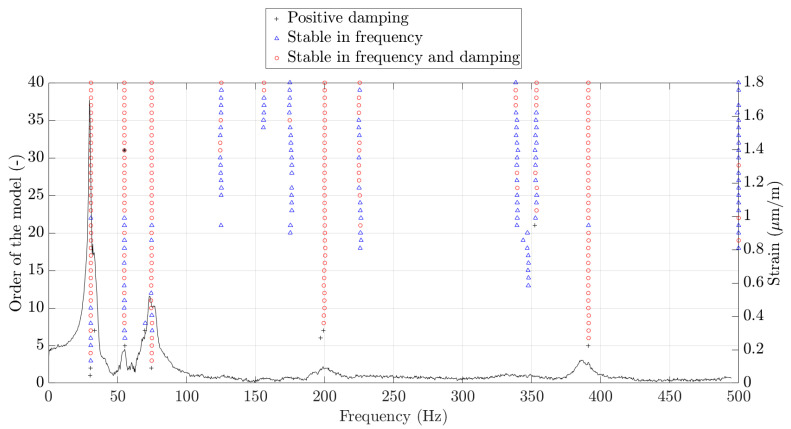
Example of stability plot for FBG10 and Test 2. In red, stable solutions in both frequency and damping; in blue, stable solutions in only frequency; and in black, positive damping solutions.

For each sensor, an averaged fast Fourier transform (FFT) was performed considering each repetition. Then, an inverse Fourier transform was performed on the resulting spectrum to obtain an averaged impact time series for each of the 19 FBGs in the array. This matrix was then used in the ERA technique, fixing the number of modes (order of the model) during the whole process. To estimate the modal parameters, stability plots were used for all FBG sensors, as Figure 10 shows for the case of FBG10 and Test 2. In the figure, the averaged FFT of the strain is displayed together with some symbols indicating the stability of each solution obtained with a different number of modes of the ERA.

Regarding the analysis of the transient tests during a ramp-up of the shaft from 0 to 200/300 rpm, a short-time Fourier transform (STFT) of an FBG sensor was performed with a frequency resolution of 0.1 Hz. A Hanning window with an FFT block size of 10,000 points was applied to compute the STFT with a sliding time window with an overlap of 99% between two consecutive FFT blocks.

## 3. Results

### 3.1. Results Related to Objective 1

**Figure 11:**
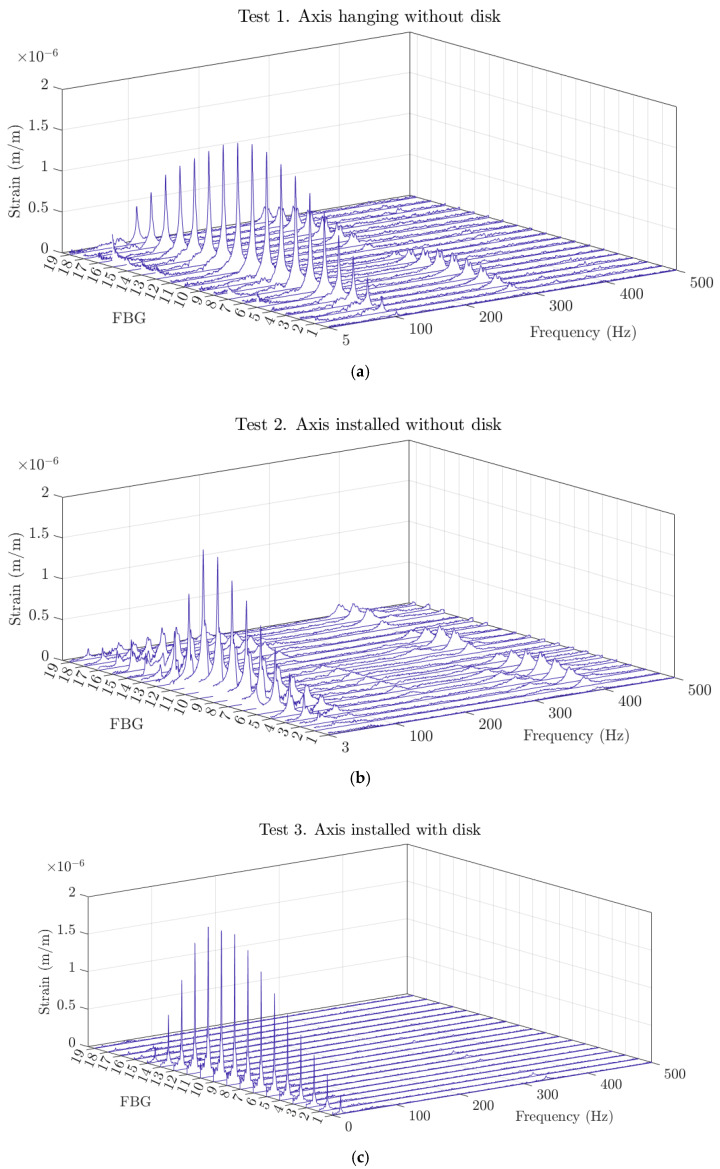
Waterfall spectra of the 19 FBG sensors during one impact test of (**a**) Test 1, (**b**) Test 2, and (**c**) Test 3.

The results obtained in Tests 1 to 3 are summarized in Table 4. Due to the large amount of data created by the 19 sensors of the FBG array, the results are given as a mean value and two times the standard deviation of the equally weighted samples from all sensors. The authors acknowledge that the samples of a distributed sensing technology should not be weighted equally when measuring the natural frequency and damping of a particular mode of vibration, as the sensors are installed in a fixed position along the shaft. This is a valid argument, but it is important to repeat here that the objective of this study is to assess the performance of the sensors and equally weighted samples to allow us to better judge how dispersed the results are and see how the results degrade/improve with sensor location. Figure 11 shows the waterfall spectra of all the sensors for the different tested conditions.

In the previous figure, it can be seen how the different boundary conditions significantly affect the FFT of each fiber-optic sensor, changing the natural frequencies of the most significant strain peaks and the whole spectra. For all three tests, a clear pattern of strain can be seen, where the zone around FBG10 has the highest strain, and this decreases as it approaches the extreme FBG sensors at the tip and the bottom of the shaft. This behavior is related to the first bending mode of the shaft, which will be further studied, in detail, in the following objective.

### 3.2. Results Related to Objective 2

For the second objective, it was decided to limit the analysis to only pure bending modes of shaft vibration. To do so, the phase of the spectra of the 19 FBG sensors was calculated for all the identified frequencies, and the mode shapes were estimated. Table 5 shows the identified frequencies and damping ratios of the first, second, and third bending modes ($f_{1}$, $f_{2}$, and $f_{3}$, respectively). It must be noted that the same fixed order was used in the analysis for all the positions of the present document, but even so, for the boundary condition corresponding to Position 1, the second bending mode, $f_{2}$, was not detected.

Once the natural frequencies of the shaft are identified, their mode shapes can be known by plotting their magnitude-phase pair. Thanks to the large number of sensors in a relatively short length of the shaft, the spatial resolution is high, and the shapes are well captured.

**Figure 12:**
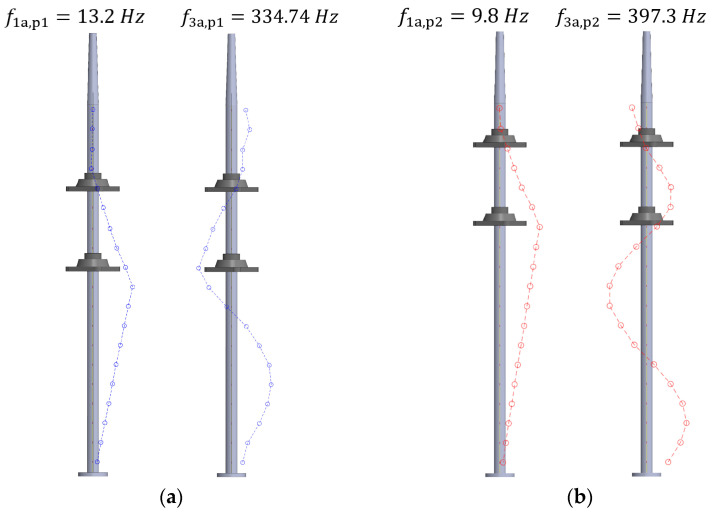
Strain mode shape comparison for the first and third bending modes in the air for (**a**) Position 1 and (**b**) Position 2.

Figure 12 shows a comparison between the first and third bending modes of the shaft for Position 1 (Figure 12a) and Position 2 (Figure 12b), both in air. It can be seen how, depending on the positions of the bearings, the natural frequencies and the shape of the strain modes change, as the strain field along the shaft is influenced by the most rigid zones around the bearings. However, due to the large number of sensors present within an FBG array, the overall pattern of the mode shape is still captured and can be correlated with the characteristics of the different boundary conditions.

**Figure 13:**
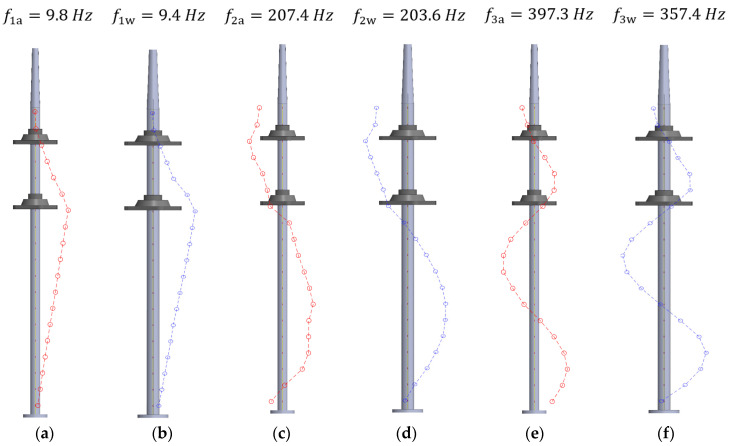
Strain mode shapes of (**a**) the first bending mode in air and (**b**) water; (**c**) the second bending mode in air and (**d**) water; (**e**) the third bending mode in air and (**f**) water.

On the other hand, Figure 13 shows the influence that the surrounding water has on the detected mode shapes of the shaft. Thus, a comparison of all three bending modes of the shaft when it is placed in Position 2 is presented when it is vibrating in the air (red line) and in water (blue line). In this case, the mode shapes are also well captured in the presence of water, where other traditional sensors may be unable to work properly. Additionally, the added mass effect of the surrounding water of the shaft can also be quantified, as there is a slight decrease in the natural frequencies when the shaft is in water.

Concretely, Figure 13a shows how the first bending mode in the air has the maximum strain around the lower bearing, which decreases linearly toward both the base of the shaft and toward the upper bearing. When submerging the shaft in water, the mode shape presented in Figure 13b has very similar behavior. For the second bending mode, appearing around $f_{2a}=207.4\;Hz$ in air, the strain mode shape has two nodes at the base of the shaft and around the lower bearing, as shown in Figure 13c. Figure 13d shows the second bending mode detected when the shaft was submerged in water, presenting the nodes approximately at the same positions. Finally, the nodes of the third bending mode are detected around the sensors FBG7, FBG12, and FBG17 in both air and water, as shown in the plots of Figure 13e,f.

It is important to repeat here that the mode shapes shown in Figure 12 and Figure 13 are strain modes. The actual modes of vibration (motion plots) could be obtained by double integrating the strain shapes. From an engineering perspective, both magnitudes are relevant for the safe operation of a machine shaft.

### 3.3. Results Related to Objective 3

**Figure 14:**
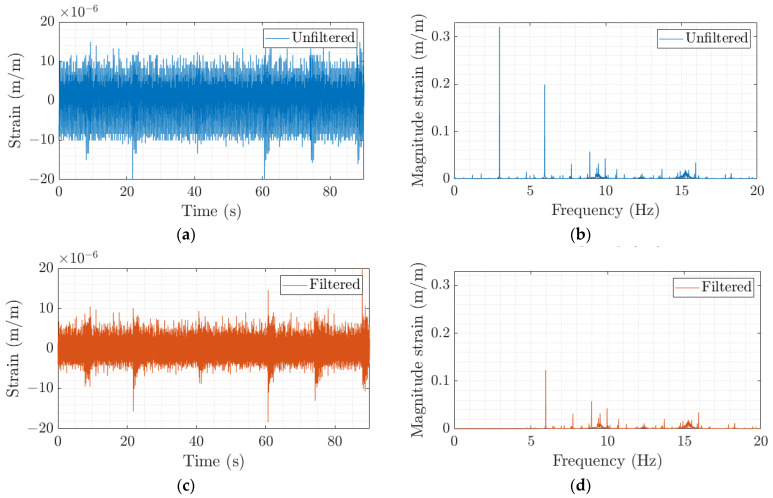
(**a**) Unfiltered time signal and (**b**) FFT spectrum of the FBG7 impacts to the shaft while rotating at 180 rpm and (**c**) highly filtered time signal and (**d**) its corresponding FFT spectrum.

Finally, for the third objective, several modal tests were performed while the shaft was rotating at 60, 180, and 300 rpm and also at rest (0 rpm). The same analysis as before was performed with these tests, except for the case of rotation at 300 rpm, which was particularly challenging for the ERA algorithm. In this case, the order was maintained, but the time series for the free decay was reduced. For this objective, it was decided to focus exclusively on the first natural frequency. It is important to stress here the difficulties in performing a modal test with an instrumented hammer on a rotating shaft to obtain good-quality data. The rotation will make the impact more challenging (and potentially risky) and will also reduce the signal-to-noise ratio (SNR) in the sensors. Figure 14 below shows an example of the obtained time series from FBG7 with six impacts while the shaft was rotating at 180 rpm. In orange, the same time series, but highly filtered to remove the rotating frequency, is displayed, in which even though the impacts are more clearly distinguishable, the SNR is still quite poor.

Table 6 shows the obtained natural frequencies for the different rotational speeds and a different configuration for the bearings (Position 3). Again, the values shown correspond to the mean frequency of all 19 FBG sensors with two times the standard deviation. It must be noted that even though the array detects the apparent change in frequency with rotational speed, it, in general, only detects the increasing frequency. Very few FBG sensors were able to capture both split frequencies at the same time. Usually, FBG numbers 9 and 10 were the only sensors in the array capable of detecting both. These sensors were located close to the lower bearing, for this particular position, and, therefore, experienced higher strains.

**Figure 15:**
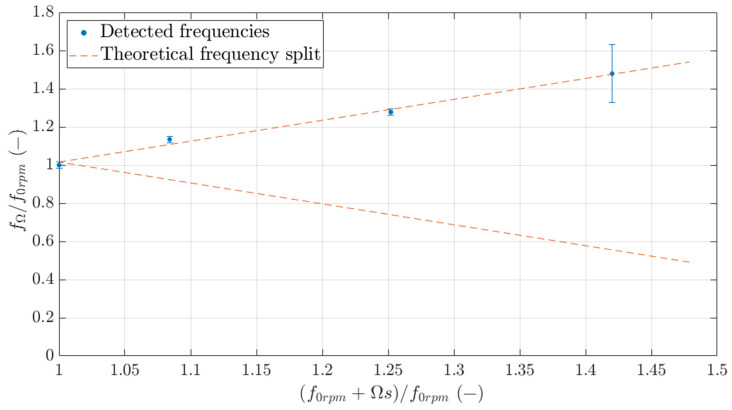
Theoretical frequency split of the first natural frequency (in orange) and detected frequency split (in blue).

Figure 15 shows a graphical representation of the averaged detected frequencies by all 19 FBG sensors (blue points) and the theoretical frequency split that should be detected according to Equation (1) (dashed orange lines). The fact that, in general, only one frequency is detected can be seen as the sensors underperforming, but this is likely due to the quality of the input data and the chosen analysis.

To ensure that the fiber sensors are effectively able to see the frequency split correctly, several transient tests were performed with another bearing configuration (Position 4).

**Figure 16:**
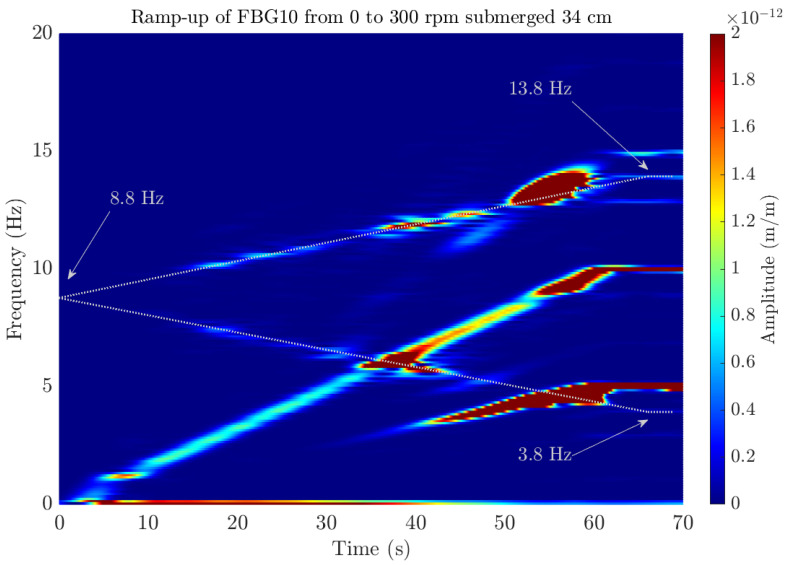
STFT of the FBG10 from a transient test where the shaft rotational speed is increased linearly from 0 to 300 rpm over 60 s. White dashed lines indicate the increasing and decreasing frequency shift detected, where the initial and final detected frequencies are marked.

The ramp-up transient tests in Position 4 were performed increasing the shaft rotational speed from 0 to 300 rpm, quantifying the frequency change over time through the STFT of the FBG10 sensor. Figure 16 shows the STFT when the shaft was submerged 34 cm inside the tank of water and a ramp-up between 0 and 300 rpm was performed over 60 s. The amplitude of the peaks in m/m is presented as a colormap ranging from the lower values in blue to the higher ones in red. Thus, the evolution of the peaks of the spectra of the FBG10 sensor can be identified by looking at the colored zones. For instance, the increasing linear line that goes from 0 to 10 Hz in 60 s corresponds to the second harmonic of the rotation, which has a frequency of 5 Hz. The frequency split of the first bending mode, marked with a white dashed line, can also be seen starting at 8.8 Hz when the shaft is stopped. In the end, when the shaft rotates at 300 rpm (5 Hz), the theoretical frequency split of Equation (1) matches perfectly with the experimental results, detecting a frequency of 3.8 and 13.8 Hz (marked in the figure). This also explains why modal analysis at 300 rpm was so challenging for the ERA algorithm, as both the decreasing and increasing frequencies of 3.8 and 13.8 Hz lay very close to the rotational frequency of 5 Hz and its third harmonic at 15 Hz, respectively.

**Figure 17:**
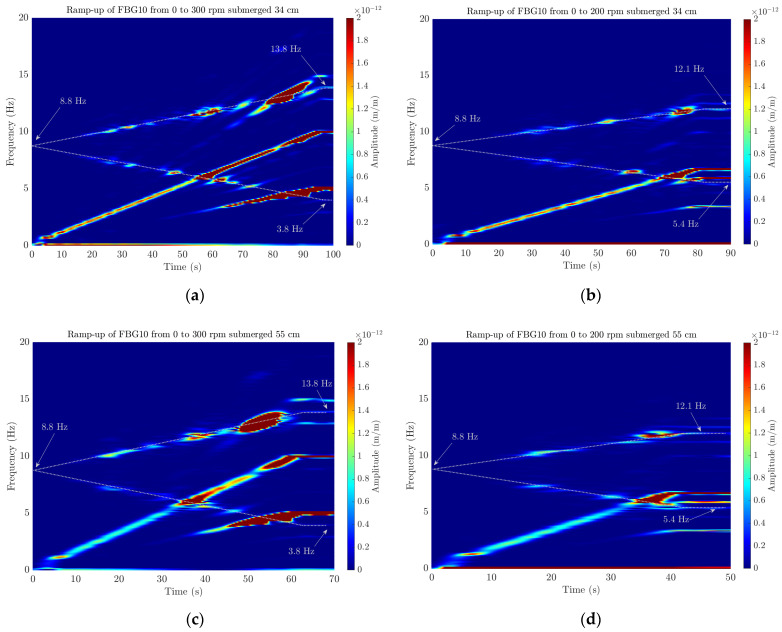
STFT of the FBG10 computed for the transient tests where the shaft was submerged 34 cm in water and the rotation was increased linearly (**a**) from 0 to 300 rpm and (**b**) from 0 to 200 rpm and for the case of being submerged 55 cm and increasing (**c**) from 0 to 300 rpm and (**d**) from 0 to 200 rpm. White dashed lines indicate the increasing and decreasing frequency shifts.

This behavior was also analyzed for other transients with different boundary conditions, where a different height of water around the shaft was considered and different ramps were performed with different durations. Figure 17 shows a comparison between the different STFTs computed for the transient with different boundary conditions. On the one hand, when the shaft is submerged 34 cm in water (Figure 17a,b), the FBG10 is able to detect the frequency split for different ramping velocities, which also matches the frequency split of Equation (1) at 300 rpm (±5 Hz) and at 200 rpm (±3.33 Hz). Additionally, by comparing these STFTs with the ones obtained with the shaft submerged 55 cm in water (Figure 17c,d), it can be concluded that, for both depths, the effect of water surrounding the shaft is the same for this mode. For all cases, the detected natural frequency at 0 rpm is 8.8 Hz, which corresponds to the first shaft’s natural frequency at rest.

**Figure 18:**
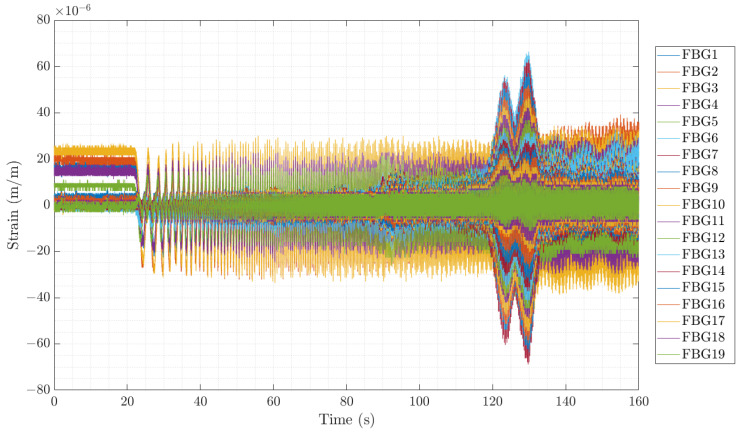
Time series showing the temporal evolution of the transient test analyzed in Figure 16.

Along the evolution of the first bending frequency marked with the white dashed line, different red zones appear in the middle of the ramp-up, which indicates a higher peak of the spectra at that time instant, corresponding to a resonance of the shaft when the rotating velocity or its harmonic excites that mode. This resonance phenomenon can also be detected in the time signal of one test (see Figure 18). The large and sudden strain increments in all the FBG sensors at the time instant between 120 and 140 s are the result of the increasing frequency meeting the third rotational harmonic and the decreasing frequency meeting the rotational frequency. Note that the strain levels increase by a factor of 2 and 3, showing the importance of monitoring this phenomenon to guarantee the shaft’s (and other components’) structural integrity.

## 4. Conclusions

The authors proved the feasibility of installing a fiber-optic array along the shaft line of a rotating structure by means of machining a longitudinal groove and embedding the fiber-optic array using epoxy glue to pass it through the bearings. Additionally, due to the rotary joint used at the top of the shaft, the optical fiber was able to withstand high rotational speeds and measure the on-board strain of the shaft in real-time while rotating.

Compared to other traditional sensors that could be mounted on-board, the installed fiber-optic sensors have the advantage of being able to monitor in locations where traditional sensors would not have worked properly due to the presence of bearings. They also performed well under different conditions, such as when the structure was submerged in water or while it was rotating. However, fiber-optic technology needs an interrogator to acquire the signals, which determines the sampling rate of the acquisition. For this research article, an sm130 interrogator had a maximum sampling frequency of 1000 Hz, which proved to be a limitation for the studied shaft and its dynamic response.

Regarding the monitoring of shaft vibrations using FBG sensors, the authors were able to track the natural frequencies of the shaft at different configurations and identify the strain mode shapes by means of performing different modal analyses. Due to the high spatial resolution achieved with the 19-sensor FBG array, slight variations in the mode shapes associated with the bearing configuration were detected and the effect of the height of water around the shaft was also quantified.

Finally, when the shaft was in rotation, the theoretical frequency split of the detected on-board natural frequency was identified with the on-board strain signal of the shaft during ramp-up transient tests.

In summary, this new fiber-optic technology was successfully assessed for a reduced-scale rotating structure, which outperforms traditional sensors in terms of compact spatial resolution, ease of installation, and robustness. Hence, in future work, this type of installation along the shaft will be reproduced on a real hydraulic turbine to validate the innovative strain sensing in more complex machines where different phenomena will take place.

## Figures and Tables

**Table 1:** Test conditions to study Objective 1.

Test	Shaft Boundary Condition	Disc
1	Hanging	No
2	Installed (Position 0)	No
3	Installed (Position 0)	Yes

**Table 2:** Test conditions to study Objective 2.

Test	Shaft Boundary Condition	Submerged
4	Installed, Position 1	No
5	Installed, Position 1	Yes
6	Installed, Position 2	No
7	Installed, Position 2	Yes

**Table 3:** Test conditions to study Objective 3.

Test	Shaft Boundary Condition	Shaft Rotating Speed (rpm)	Submerged	Test Type
8	Installed, Position 3	0	No	Impact
9	Installed, Position 3	60	No	Impact
10	Installed, Position 3	180	No	Impact
11	Installed, Position 3	300	No	Impact
12	Installed, Position 3	50, 100, 150, 200, 250, 300	Yes	Monitor
13	Installed, Position 3	50, 100, 150, 200, 250, 300	Yes ^1^	Monitor
14	Installed, Position 4	From 0 to 200	Yes ^2^	Monitor
15	Installed, Position 4	From 0 to 300	Yes ^2^	Monitor

^1^ Difference between Tests 12 and 13 is the volume of water in the tank. ^2^ Tested at two different submergence levels of 34 and 55 cm.

**Table 4:** Averaged natural frequencies and damping ratios of all FBGs related to Objective 1.

	Test 1	Test 2 (Position 0)	Test 3 (Position 0)
$f_{1}(Hz)$	101.5 ± 0.1	30.3 ± 0.3	12.3 ± 0.1
$f_{2}(Hz)$	284.5 ± 0.3	74.7 ± 0.4	51.2 ± 0.0
$f_{3}(Hz)$	435.5 ± 0.8	391.4 ± 0.2	334.8 ± 0.1
$\xi_{1}\left(\%\right)$	0.9 ± 0.1	4.4 ± 1.4	2.8 ± 0.3
$\xi_{2}(\%)$	0.8 ± 0.1	3.5 ± 0.5	1.4 ± 0.0
$\xi_{3}(\%)$	1.0 ± 0.2	0.7 ± 0.1	0.4 + 0.1

**Table 5:** Averaged natural frequencies and damping ratios of all FBGs related to Objective 2.

	Position 1		Position 2	
	Air	Water	Air	Water
$f_{1}(Hz)$	13.2 ± 0.2	12.2 ± 0.1	9.8 ± 0.1	9.4 ± 0.1
$f_{2}(Hz)$	-	-	207.4 ± 0.6	203.6 ± 0.2
$f_{3}(Hz)$	334.7 ± 0.4	326.0 ± 0.1	397.3 ± 0.7	357.4 ± 0.5
$\xi_{1}\left(-\right)$	4.02 ± 0.83	4.29 ± 0.15	3.80 ± 0.21	4.08 ± 0.31
$\xi_{2}(-)$	-	-	1.23 ± 0.11	0.82 ± 0.07
$\xi_{3}(-)$	0.92 ± 0.12	0.92 ± 0.06	0.56 ± 0.08	1.20 ± 0.20

**Table 6:** Averaged first natural frequencies of all FBGs for the different tested rotational speeds.

	0 rpm	60 rpm	180 rpm	300 rpm
$f_{1}(Hz)$	11.9 ± 0.2	13.5 ± 0.2	15.2 ± 0.2	17.6 ± 1.8

## Data Availability

Not applicable.
